# Artificial Intelligence and Big Data in Diabetes Care: A Position Statement of the Italian Association of Medical Diabetologists

**DOI:** 10.2196/16922

**Published:** 2020-06-22

**Authors:** Nicoletta Musacchio, Annalisa Giancaterini, Giacomo Guaita, Alessandro Ozzello, Maria A Pellegrini, Paola Ponzani, Giuseppina T Russo, Rita Zilich, Alberto de Micheli

**Affiliations:** 1 Italian Association of Diabetologists Rome Italy; 2 Diabetology Service, Muggiò Polyambulatory, Azienda Socio Sanitaria Territoriale Monza Italy; 3 Diabetology, Endocrinology and Metabolic Diseases Service, Azienda Tutela Salute Sardegna-Azienda Socio Sanitaria Locale Carbonia Italy; 4 Departmental Structure of Endocrine Diseases and Diabetology, Azienda Sanitaria Locale TO3 Pinerolo Italy; 5 New Coram Limited Liability Company Udine Italy; 6 Operative Unit of Diabetology, La Colletta Hospital, Azienda Sanitaria Locale 3 Genova Italy; 7 Department of Clinical and Experimental Medicine, University of Messina Messina Italy; 8 Knowledge Management - Mix-x Milano Italy; 9 Associazione dei Cavalieri Italiani del Sovrano Militare Ordine di Malta Genova Italy

**Keywords:** artificial intelligence, big data analytics, clinical decision making, diabetes management, health care

## Abstract

Since the last decade, most of our daily activities have become digital. Digital health takes into account the ever-increasing synergy between advanced medical technologies, innovation, and digital communication. Thanks to machine learning, we are not limited anymore to a descriptive analysis of the data, as we can obtain greater value by identifying and predicting patterns resulting from inductive reasoning. Machine learning software programs that disclose the reasoning behind a prediction allow for “what-if” models by which it is possible to understand if and how, by changing certain factors, one may improve the outcomes, thereby identifying the optimal behavior. Currently, diabetes care is facing several challenges: the decreasing number of diabetologists, the increasing number of patients, the reduced time allowed for medical visits, the growing complexity of the disease both from the standpoints of clinical and patient care, the difficulty of achieving the relevant clinical targets, the growing burden of disease management for both the health care professional and the patient, and the health care accessibility and sustainability. In this context, new digital technologies and the use of artificial intelligence are certainly a great opportunity. Herein, we report the results of a careful analysis of the current literature and represent the vision of the Italian Association of Medical Diabetologists (AMD) on this controversial topic that, if well used, may be the key for a great scientific innovation. AMD believes that the use of artificial intelligence will enable the conversion of data (descriptive) into knowledge of the factors that “affect” the behavior and correlations (predictive), thereby identifying the key aspects that may establish an improvement of the expected results (prescriptive). Artificial intelligence can therefore become a tool of great technical support to help diabetologists become fully responsible of the individual patient, thereby assuring customized and precise medicine. This, in turn, will allow for comprehensive therapies to be built in accordance with the evidence criteria that should always be the ground for any therapeutic choice.

## Introduction

The ongoing evolution in medicine and, in particular, in the field of diabetology is strongly intertwined to a series of changes and innovations [1-3]. The term “digital health” is a “container,” grouping together informatics and telecommunications that have the common objectives of diagnosis, treatment, or monitoring of diseases, maintenance of health and well-being, and support for healthy lifestyles. The US Food and Drug Administration has compiled a list of software programs with medical device functions (apps for smartphones and personal computers with diagnostic, monitoring, or therapeutic objectives), advanced business intelligence data analysis tools, artificial intelligence (AI), cloud, cyber security, and innovative health technologies [4]. These tools belong to an evolving reality; they are still unclear but they are potentially leading to challenging and promising scenarios [5,6].

The scope of this position statement is to analyze the most relevant aspects and describe the changes that have already occurred or those that will take place shortly by exploring the possibilities of application and development in the field of diabetology. Indeed, the digital world is constantly expanding and it has already become an integral part of our personal and professional life [7,8]. Smartphones, personal computers, and network access are now essential tools for almost all populations and for many diabetologists, both as individuals and professionals.

### Digitalization

Almost every daily activity of the diabetologist has to deal with digitalization, from electronic medical records to imaging diagnostics, from laboratory references to the various software programs for administrative practices and certifications, and from glucose level data downloaded from glucometers to sensors for continuous glucose monitoring and insulin pumps. Through the analysis of these data, the diabetologists make therapeutic decisions, and the more complete and numerous the data, the more they need informatics tools to guide them in the analysis, help them identify specific patterns for detecting glycemic abnormalities, understand the possible causes, and adopt the appropriate therapeutic strategies to correct these abnormalities [9,10].

### Data Management and Connectivity

In the early days, data collection and management for each individual patient occurred manually at the hospital by physically connecting cables or wireless devices to the local software/network. Currently, thanks to the technological evolution, these data can be transmitted automatically from the glucometers via “cloud” or on data integration platforms that collect elements from different devices and provide standardized reports [11,12]. If not properly managed, this large amount of data is likely to overwhelm both the patient and the health care professional, and the professionals’ experience in reading these reports is certainly the added value that increases their potential. Advanced informatics tools can simplify the analysis and provide suggestions to guide the clinical decisions of the physician [13]. With experience and technology coming together, the analysis time can be increasingly reduced and more appropriate and correct data-driven therapeutic decisions can be made, thanks to the greater customization strategies of the therapy [14-21].

### AI and Individualized Care

In this context, the use of AI, and in particular, the use of machine learning allows an important step forward compared with the traditional data analysis techniques (ie, graphs representing a picture of reality, which are very useful and precise but are static and outdated). Through the automatic identification of specific patterns within the data and through inductive reasoning typical of the human mind, machine learning can highlight correlations that lead to “predictions,” without being programmed in advance to carry out this activity [19].

In a not too distant future, AI, thanks to algorithms that can enable learning and improving the machine’s own abilities independently, will offer effective solutions to satisfy the most disparate needs and will be able to deal with problems that today may seem insurmountable obstacles, for the benefit of the community [22]. In the field of clinical diabetology, these tools could have multiple potential implications, including the identification of new risk factors for the onset of diabetes (through the evaluation of large databases related to the general population) or revealing unsuspected subjects who are at a high risk of complications (by cross-checking clinical and administrative records of patients with diabetes) and identifying the behavioral and therapeutic variables that are most closely related to the progression of a specific complication.

There are many examples of collaboration between pharmaceutical companies, information technology companies, scientific institutes, and universities that exploit large complex datasets (the so-called big data) with the aim of improving the treatment of type 2 diabetes mellitus (T2DM) and of unraveling physiopathological mechanisms through the integration of data from biological, demographic, clinical, environmental, and genomic sources [23].

### Risk Stratification and Personalized Medicine

The ability to elaborate a large and heterogeneous amount of data, even in real time, through increasingly powerful algorithms, allows us to extract knowledge and exponentially make predictive assessments on the behavior of individuals as well as, more generally, assume decisions for the entire community [24,25]. This means that the use of AI will make it possible to transform the immediate data (descriptive) into knowledge of the factors that “condition” behavior and correlations (predictive) [25-27] up to the identification of the key factors that can facilitate an improvement in the expected results (prescriptive) [28,29]. In this scenario, one of the most interesting applications would be to identify the variables that could be related to greater responsiveness to a specific drug because this approach would open the door to a truly personalized medicine that uses the right drug for the right person, with obviously greater efficacy, improved outcomes, and containment of costs. Furthermore, predictive analysis techniques based on AI could be used to identify which group of patients would require more attention and which strategies would be more effective, depending on the individual patient, thereby allowing more efficient methods of personal care with lower costs and better outcomes. This concept of risk stratification, which considers all the individual characteristics of the patient (ie, clinical aspects, genetic data, lifestyle, environmental factors), is the basis of the modern model of clinical governance called “population health management.” Today, the risk stratification of the population results from the extraction of historical expenditure data (eg, inpatient and outpatient admissions, diagnosis, pharmaceutical expenditure) and this presents the following series of biases: weak clinical validity; temporal misalignment between the extraction, the analysis, and the health status; and absence of information on the real socioeconomic condition and behavioral health. In this way, the data represent “satisfied demand” rather than “real needs.” The new challenge is the use of big data and business analytics systems to activate risk stratification models based on real health status, integrated use of multiple sources, and collaboration between professionals (ie, care team and data manager in a data mining process) [30,31].

### Biotechnologies and Omics

Another aspect that characterizes the ongoing medical revolution is the development and increasing importance of biotechnologies used in genetics and in the so-called “omics” sciences. These new techniques have been showing that the complex pathophysiological processes underlying type 1 diabetes mellitus (T1DM), T2DM, and gestational diabetes mellitus are caused by disturbances in the gene expression that lead to alterations in the processes within the organs involved in glucose homeostasis [32]. The complexity of the system is exacerbated by the fact that the relative contribution of each component is highly individual. Understanding the molecular mechanisms underlying these interactions is crucial for developing new personalized prevention and treatment strategies [32,33].

### Social Networks and Apps

Finally, the ever-increasing use of social networks and apps has major implications in the diabetes care. On one hand, health professionals must change the way they communicate and keep up with the times by always acquiring new technological and communication skills to deal with and manage the changes taking place, while on the other hand, the new technologies could be used as motivational and coaching tools in support of the traditional educational activities and as an alternative data source. Notably, the areas that influence the state of health are only 10% due to the medical care and health status, whereas the major role is instead played by lifestyle and behaviors as well as by genetics [34-36]. For this reason, to obtain a more precise picture of the state of health of the general population or of specific subgroups, we cannot limit ourselves to collecting and analyzing only health data, but we must consider what comes from the world of social networking and smartphone apps, despite all the known limitations and criticalities.

## Evolution of the Role of Health Care Professionals

The exponential increase in knowledge and technologies, the increased complexity of the tasks, and the increasingly diversified needs of the patients are flooding health care professionals with increasingly greater tasks and aspects to be addressed and managed. It is necessary to reflect on what the true essence of medical care is and to reassess the meaning of this profession. The ability to change, juggle the new technology tools, and exploit the potential of new information technology and business analytics techniques will enable health care professionals to have a great support in their choices and reduce the time spent collecting data or using the machines so that they can concentrate more on the decision-making process, thereby rendering each intervention more effective and efficient. As a proof of concept, it has been recently reported that the costs for care of patients were steadily diminished by the active use of technology, which supplied a real time feedback about their blood glucose levels and connected them to clinical support [37]. These reductions were primarily attributable to reduced diabetes-related and office-based services, independent of the specific condition, and they were proportional to the frequency of use of the digital tools [37].

## Data from Diabetology Literature

Diabetology is facing different challenges: the ever-decreasing number of diabetologists, the increasing number of patients, the reduction of visitation time, the increasing complexity of the pathology both from the clinical and welfare point of view, the difficulty in achieving the objectives, the growing burden of pathology management for health care professionals and patients, and the decreased accessibility to care and sustainability. New digital technologies and the use of AI are certainly a great opportunity. The current panorama of international scientific literature with respect to the use of big data and AI in diabetology offers various hints and in-depth analyses, which are applicable to different fields.

### The Epidemiological Area

Cases of diabetes have been identified within large heterogeneous databases, and new risk factors for diabetes have been identified. An interesting multi-database retrospective study on the identification of cases of T2DM [17] strategically used the European Medical Information Framework Project database, a European project for the efficient reuse of health data for epidemiological research [38]. This database collects health information of about 52 million European citizens by using heterogeneous sources and by acting as a support for the execution of high-quality multinational observational studies, based on populations with large sample sizes and otherwise inconceivable follow-ups. Subjects with T2DM were identified using a complex algorithm strategy in 8 different health data sources, and the strengths and limitations of each data source were revealed during the creation of a model that ensures the interoperability of systems of heterogeneous electronic medical records. These efforts represent a methodological advancement for carrying out studies of multinational and multi-data sources, thereby providing sufficient information for the contextualization and correct interpretation of the results and generating transparent and reusable documentation.

Additionally, a study conducted by researchers from the University of New York and Philadelphia described a new “data-driven” approach of population health management based on the use of machine learning techniques to develop predictive models and identify risk factors for the onset of T2DM [39]. They based this model on administrative data concerning health services, pharmaceutical records, insurance databases, citizens’ access to the different care facilities, and laboratory results from 4.1 million individuals over a period of 4 years and a total of 42,000 variables. This model has been able to identify new risk factors for the appearance of diabetes with a predictive probability of at least 50% higher than the models based on the known traditional risk factors, thereby avoiding the costs of performing a screening. The identification through new predictive models based on machine learning of the part of the population that is at the highest risk of developing diabetes will be able to generate clinical hypotheses for the identification of new risk factors and to implement more targeted cost-effective interventions.

### Phenotyping and Risk Stratification

A study of 65,000 newly diagnosed patients with T2DM [40] estimated that 10% of the subjects absorb 68% of the resources. The task of predictive analytical methods based on big data is precisely that of identifying the 10% of the subjects most at risk, on whom the treatment needs to be intensified in order to improve the health outcomes at lower costs.

An interesting Japanese study [41] has developed a practical framework for phenotyping T2DM by using both specialized knowledge and an approach based on machine learning (in particular, the support vector machine) to develop 2 phenotyping algorithms with data extracted from electronic medical records: one with high sensitivity for screening and the other to identify the subjects for research. Both algorithms showed superior performance compared to the basic algorithms, thus suggesting the possibility of using the proposed framework to conduct appropriate research depending on the objective.

### The Diagnostic Area

The use of predictive models based on AI has demonstrated the feasibility of identifying individuals with the highest probability of having undiagnosed diabetes through clinical data that can be easily obtained from different databases [42], thereby exploiting the potential of machine learning algorithms, including neural networks, as tools for diagnosing diabetes [43]. Machine learning and pattern recognition are tools of enormous interest as they are promising in improving the sensitivity and specificity of disease detection and diagnosis, and they appear to be able to reduce the potential for human error in decision-making. An exemplar application is the creation of surveillance algorithms that are able to detect diabetes and, in particular, to distinguish T1DM from T2DM by using structured electronic medical records [14]. The extraction of data from the electronic database of a 4-year long, multi-sectorial, and multi-specialist outpatient practice allowed the inclusion of approximately 700,000 patients. Possible cases of diabetes were reported using laboratory test results, diagnostic codes, and prescriptions. More cases of diabetes were captured by taking advantage of the entire range of data acquired from the records compared to that captured by the analysis of only administrative databases, thus increasing the sensitivity of the method. The application of these algorithms to electronic folders has the potential to provide timely and clinically detailed information on large numbers at low cost and nearly in real time. Electronic records will probably become increasingly important sources for the surveillance of public health and for the definition of more targeted health policies [15,44].

### Field of Automatic Reporting

Diabetic retinopathy, in particular, is a chronic complication of diabetes. The automated classification of diabetic retinopathy has potential benefits such as increasing the efficiency, reproducibility, and coverage of screening programs; reducing obstacles to accessing and improving results; and providing early diagnosis and treatment. To maximize the clinical utility of the automated classification, an algorithm designed to detect specific lesions or to predict the presence of any level of diabetic retinopathy was recently developed [45]. In this study, deep learning, together with visual and pattern recognition techniques, has allowed the identification of the desired features with the highest predictive value directly from the images on a large set of labeled example data. These results show that deep neural networks can be “trained” by using large data sets and without having to specify lesion-based features to identify diabetic retinopathy or diabetic macular edema in fundus images with high sensitivity and high specificity.

### Economic Field

Cost-effectiveness studies of health interventions have been performed previously. An Italian group performed a retrospective analysis through the cross-examination of large clinical and administrative databases with the aim of quantifying the relationship between health care costs attributable to diabetes and the level of glucose control [46]. The results indicated that glycemic control (expressed by hemoglobin A_1c_ [HbA_1c_] levels) is a useful surrogate not only to estimate the odds of developing diabetes-related complications but also to estimate the costs associated with health care. The integration of administrative and clinical databases seems to be suitable to show that an appropriate management of diabetes can allow a better allocation of resources.

### Expected Advantages in Diabetology

The ongoing medical revolution is strongly linked to the spread of digital health, the new software for AI, the use of big data to make more appropriate data-driven decisions, and the even greater focus on predictive, preventive, personalized, and participatory medicine [6]. Each of these elements has important repercussions in the management of complex and widespread chronic diseases such as diabetes. Table 1 summarizes the advantages and weaknesses attributed to each area of application, especially considering the challenges that diabetology faces, according to our professional experience and opinion.

**Table 1:** Advantages and weaknesses of the use of new technologies in diabetology (our opinion).

New technology in diabetology	Advantages	Issues
Digital data management (glucometers and continuous glucose monitoring connected to the cloud and data integration platforms)	Support for doctors’ decisions Reduced analysis time Graphs and images easy to understand and interpret Correct management, supported by data, even remotely Sharing with caregivers or family members possible Simultaneous analysis of data from different devices Integration of glycemic values with alternative data for better understanding (eg, carbohydrate intake, physical activity) Possibility of intervening in the intervals between visits Overcomes geographical barriers Motivational tool	Difficult to integrate with computerized clinical records Different software programs for different devices Time spent learning the software and gaining experience Risk of data “flooding” the professional and the patient Lack of significant evidences on the improvement of the outcomes Limited number of patients currently accessing this technology Lack of recognition for time spent and medical services Requirement of organizational changes
Mobile app (medical device with CE^a^ marking)	Therapeutic instrument (eg, bolus calculator) Easy visualization of data and management of corrective actions Overview of trends over time Greater patient involvement Convenient for the patient Motivational and educational support tool	New skills and time for patient training Reliability of the instruments
Telemedicine	Overcomes geographical barriers Greater accessibility to care Reduced administrative burden (if structured) Lower costs and inconvenience to the patient Integration with traditional management in the clinic Strong potential for cost reduction	Nonrecognition of medical services Structural difficulties Need for institutional and organizational changes
Machine learning	Performance of descriptive, predictive, and prescriptive analyses Analysis of large databases of different sources that cannot be analyzed with traditional statistics Better epidemiological risk assessment of the disease Identification of new variables and new risk factors for the development of diabetes and its complications Possibility of identifying the most effective patient-tailored therapeutic strategy Minimizing adverse drug events by increasing safety Possibility of phenotype/genotype integration	Data quality Heterogeneity of unstructured data Correct use of data Integration of data from different sources Respect for privacy Ethical problems Possibility of reducing the professional skills of doctors Replacing the professional with the machine Difficulty in knowing and interpreting new analysis models different from the traditional clinical epidemiology of evidence-based medicine

^a^Conformité Européene.

## Importance of the Chronic Care Model

A very interesting field of application concerns the Chronic Care Model (CCM), a medical assistance model for patients with chronic diseases. The CCM is a model for sustainable chronicity [47], in which the scope is to achieve a “personalized and effective care,” with an active participation of the person, integrating the different professionals involved in assistance, to concretely improve the life of the person with a chronic pathology. The CCM in the declination of its various dimensions (health care organization, delivery system design, decision support, clinical information systems, self-management support, and community resources) brings a huge amount of the so-called “real world data.” Such real world data are data collected in the normal clinical practice (not from controlled clinical trials), which allow the description of the patient’s care pathways through the integration of different sources, consistent with what usually happens in reality. Each step of the care and assistance pathway—from diagnosis to treatment and follow-up—generates a large amount of data and images (big data) that often reside within health facilities in separate and independent databases. To obtain an integrated view of the diagnostic-therapeutic pathways for individual patients and to trace their complexity, it is necessary that these flows are integrated.

An interesting Australian study on CCM [48] shows how the use of electronic medical records and informatics-integrated assistance can improve the management of chronic diseases. The benefits for health care professionals and service users through an accurate and timely exchange of information are better work efficiency, prevention of repetition of data and information collection, as well as a better decision-making process.

## Perspective of AMD

In Italy, in total, about 5 million people have diabetes (about 1 in 12 residents), and the number will probably rise to 7 million in 15-20 years. Italian epidemiological data suggest that around 250,000 new diagnoses of T2DM and around 25,000 new diagnoses of T1DM are reported each year [49]. Since the 1970s, an articulated network of diabetes centers has developed—some operating independently as simple, complex, departmental, or territorial structures, while others with outpatient activities are attached to the Internal Medicine and Geriatrics Operative Units. The social and health relevance of diabetes has been sanctioned by a law (n. 115 of 1987) that has enhanced the role of diabetes centers and inspired numerous national and regional documents for the following 30 years and the approval of the National Diabetic Disease Plan in 2013 by the Ministry of Health. This model encompasses family doctors and a widespread network of specialist centers throughout the national territory, based on multi-professional skills (eg, diabetologist, nurse, dietitian, sometimes psychologist or podiatrist, and eventually cardiologist, nephrologist, neurologist, ophthalmologist) and regularly serving about 50% of the people with diabetes. As a result of this network, Italy has the lowest average level of HbA_1c_ and the lowest rates of chronic complications and excess mortality among the western countries [50,51]. In this regard, the role of diabetes care in reducing mortality in people with diabetes must be underlined; those who are assisted in diabetes centers have lower total and cardiovascular mortality than those who do not attend the diabetes centers [50,51].

The mission of the Italian Association of Medical Diabetologists (AMD) is to promote the professional development of its members and to ensure the continuous improvement of the quality of assistance to all people with diabetes. In ancient times, AMD had already grasped the importance of standardizing the recording of daily clinical work in an electronic folder, followed by the collection and processing of real life information on care, examinations, complications, and therapy for a qualitative interpretation of real assistance in the national territory. This intuition of the use of variables with an intrinsic clinical and professional integrated meaning, which imply the doctor’s reasoning in predictive and prescriptive choices has in fact realized the first model of decisional learning, thanks to the use of statistical algorithms.

### Experience of the Annals

The AMD Annals represent a periodic publication that has allowed, annually since 2006, to assess the care profiles of people with T1DM and T2DM who have been admitted in Italian diabetes care centers. A wide network of diabetology services equipped with a computerized clinical record system, used for the normal management of patients in charge, has a software program provided by AMD that allows the extraction of a standardized set of clinical information (ie, Data File AMD). The database obtained is used to calculate the quality of the care indicators, both centrally and locally. In this way, a benchmarking activity is promoted based on the comparison of one’s own performance with that recorded at the national level (best performers approach). This activity reflects the professional performance of the diabetologists—it is perfectly in line with the recommendations of the National Diabetes Plan. It is very cost-effective and has produced a systematic improvement over the years of all the indicators considered [52].

In addition, the AMD Annals database represents a valuable source of observational research data. In fact, it has allowed the investigation of many key aspects over time, such as the care of the elderly patients, gender medicine, cardiovascular, renal, and hepatic issues, the appropriateness of the use of drugs, and glycemic self-control, thereby providing a realistic picture of the work of the diabetology services. Notably, the analysis of the Annals database has allowed the identification of critical areas and, therefore, the timely activation of processes of improvement, in a logic of continuous quality enhancement, that is, a process of periodical performance assessment of diabetes centers on data collection and quality of care according to standardized indicators [53-57]. It is easy, at this point, to visualize that the Annals project “photographs” the history of the evolution of clinical diabetology and has allowed, for its own conception and structuring, each individual center to self-evaluate and improve in real time. Precisely, this careful measurement of behaviors and results is capable of triggering a dynamic evolution of an entire class of professionals, thus representing an irreplaceable benefit.

With innovative and far-sighted thinking, AMD has created this database, which today represents an unparalleled heritage in a cultural civilization that is increasingly aware of the importance of information and the value of a large and accurate collection of data. In this new era, the world is being organized to take advantage of the ever-increasing databases to rely on technology and use them as assets to be interpreted to facilitate and accelerate important decisions in every field. It is clear that it is increasingly important to have a quality data collection that is increasingly “clean,” and it is fundamental to highlight the need for all professionals to be trained in the culture of big data and its correct recording. In fact, given that decisions can be made on the results of data processing, it is essential to raise the problem of the truthfulness of the data on which the analyses are based, to have a measure of the reliability of the results, and to be aware of them. Further, in this area, AMD has invested time and resources in activating a process—born more than 10 years ago—of culture on the quality of the data, which involved many partners who participated in the Annals project [53-57]. This is why AMD feels ready to investigate the topic, as it has an advantageous background, and it wants to project itself in a competent and proactive way in the world of big data and AIs that represent a new cultural challenge in the scientific field.

### Diabetes Intelligence, the Value of the Diabetologist: Structured Skills and Prioritized Activities

The DIA&INT (Diabetes Intelligence) is an AMD project that rises in the setting of exploring new methods of analysis of unconventional data, and it can be considered as the first experiment in analyzing unstructured data by using the business intelligence method (a precursor of machine learning systems).

In a comprehensive vision of clinical activity, AMD focuses on the value of the skills that identify a diabetologist and make it a decisive tool in the care process. To achieve this objective, AMD implemented a business intelligence project called DIA&INT [47]. This project was mainly aimed at encouraging the implementation of the CCM in an “evidence-based” way by highlighting the direct link between the activities carried out by the diabetologist and their expected outcomes in order to highlight those activities that are essential to obtain the best outcomes in modern diabetology. Furthermore, this method may contribute to extrapolating the actions that could optimize the scarce resources and it may represent a valid support for the institutional choices in the current health system revision.

Admittedly, DIA&INT has been designed to respond to the need for establishing the qualitative dimension of the diabetologist’s performance and the specific contribution of the many factors participating in the clinical decision in the real world. To do this, AMD has chosen to use the following accredited tools.

1. Organizational analysis to measure and enhance the role of diabetes care with specific tools (social return on investment).
2. Data management with advanced tools (business intelligence).

The resulting “program” has been structured to standardize the information and define activities and competences, as implicitly described in the official guidelines, which are measurable and comparable, with processing methods different from those of classical epidemiology, but necessary to perform predictive and prescriptive assessments. The methodology used [47] exploited the collective intelligence of the diabetologists who participated in a survey, expressing their opinion on a complex node such as the definition of the integrated benefit of certain activities in a personalized and weighted way. The intention was to “display” how diabetologists think when carrying out clinical judgment.

Through this approach, 2 main concepts were outlined: the requirements (priority, specificity, frequency, and multidimensional analysis) and the decision-making elements for “if…then” choices based on a priori knowledge (medical, regulatory, ethical, psychosocial, etc) and the ability to propose a dynamic personalized treatment project to the person with diabetes that does not yet involve healing and that requires an active personal effort. By doing so, DIA&INT has selected the necessary skills and activities in medical practice, which have an impact on the evolution of the quality of the “health product” and the outcomes. DIA&INT produced the Core Competence curriculum of the diabetologist [58] and measured the impact of the activities on the experiences [47].

## Discussion

The changes offered by technological innovation have generated an unprecedented level of data collection and processing that is destined to undergo further expansion with the new applications of robotics and augmented reality, crossing a new frontier and entering the era of big data and cognitive systems. A new category of technologies is born, which uses natural language processing and machine learning and is able to amplify and accelerate the digital transformation process to allow people and machines to interact in a more natural way, extending and enhancing cognitive skills and abilities. The possibility of extracting information that has a meaning and is functional, in fact, requires the development of sophisticated technologies and interdisciplinary skills to operate closely together. In particular, in medicine, health care systems require consistent, appropriate, and sustainable choices.

Today, the complexity of medicine certainly goes beyond the capacity of the human mind. The patients themselves are increasingly complex and we know that the long-term effectiveness of a treatment depends on variables that are no longer just “numerical,” but also on other information that is difficult to structure [2]. In this framework, advances in computing power play a central role in the acquisition of knowledge. It is essential to collect and use the key information in a coherent way, out of its abundance by using effective and reliable analysis tools that are represented by the new techniques of AI already available today. These techniques recognize and use machine learning systems that are able to “extricate themselves” and learn from these immense amounts of data, even with intrinsic systems of recognition and error management. In essence, AI is a machine capable of solving problems and reproducing activities typical of human intelligence [59].

However, at this stage, it is important to remind our readers that even though the data are processed through algorithms, the final decision rests with the physician, and the fact that still a huge part of this category is reluctant to computerization should not be overlooked. In addition, more research is needed to assess how clinicians currently incorporate digital tools into their practice and the prevalence of digital platform use in various health care systems. AMD has already set the basis of culture and tools on the importance of collecting data, but now, it is necessary to go further. The strong potential of the large amount of data collected in more than 10 years of observation in the AMD Annals initiative may contain a significant “hidden” knowledge that, analyzed through different tools, may show new patterns that can help us to make better decisions in the prevention and management of diabetes and its complications. For example, this type of analysis may not only amplify our notions on the risk factors for diabetes complications but may also unravel new and unsuspected connections between them, indicating the probability with which they can affect the evolution of the disease on specific groups of subjects. Properly “trained” machine learning algorithms are able to efficiently evaluate millions of data while seeking probabilistic risk correlations, and they are not limited to tracking the spread of an epidemic but has the scope of identifying new personalized therapies. We could also imagine going further, in the increasingly realistic hypothesis of linkage between the various health care databases (administrative data, assistance process, intermediate and final results, costs, etc), which will allow us to make comprehensive assessments of the whole process of care in an even more individualized way in terms of clinical effectiveness, organizational effectiveness, sustainability, and equity.

The field of big data is already a reality and this systematic revision and position statement was made to offer the basis of knowledge for a constructive debate on the next steps that the AMD as an association and, more in general, diabetology would follow in the very next future. It is time to choose to be proactive players in this complex system and take responsibility for these new processes aimed at improving the care of people with diabetes, which is and remains our “mission.”

In conclusion, the incessant progress of these changes brings into question many established paradigms, also in the scientific sphere, and this position statement represents the first document of careful analysis of this new world, which we must make our own in a logic of constructive comparison. We must reflect on the current scenarios, ask ourselves about the effects produced by these transformations to understand the consequences in our lives induced by the automated decisions, and become able of integrating the traditional route into the new systems.

The diabetologist, more than any other health care professional, already has the right mentality of being ready to take up this innovation. The culture of the data is in our DNA, the need to phenotype the patient and personalize the care and therapeutic approach have long been our priorities, the skills to manage a complex disease such as diabetes have been refined over time—ranging from technological to communicative and from educational and andragogical to managerial abilities. Thanks to all this progress made over the years and strongly desired by AMD, we are ready for a new challenge in the management of diabetology that must see us as protagonists. The “digital diabetes” is coming, and AI and big data are opening a window on new scenarios. Today’s diabetologists must acquire new skills to be able to lead these changes, be proactive in exploiting their potential and advantages, and limit their risks and guard the essential elements of the professions of doctors and health care professionals managing patients with complex chronic conditions.

## Abbreviations

AI: artificial intelligence
AMD: Association of Medical Diabetologists
CCM: chronic care model
DIA&INT: diabetes intelligence
HbA1c: hemoglobin A1c
T1DM: type 1 diabetes mellitus
T2DM: type 2 diabetes mellitus
